# Immunoembolization in liver-predominant metastatic uveal melanoma: a single-center retrospective analysis

**DOI:** 10.3389/fonc.2026.1752725

**Published:** 2026-04-23

**Authors:** Renee Morecroft, Jordan Phillipps, Amrat Kumar, Jacob Strelnikov, George Nassief, Naganathan Mani, Jennifer Gould, Tanner Johanns, George Ansstas

**Affiliations:** 1 Department of Internal Medicine, Hospital Corperation of America (HCA) Florida Orange Park Hospital, Orange Park, FL, United States; 2 Department of Dermatology, Mayo Clinic Florida, Jacksonville, FL, United States; 3 Division of Medical Oncology, Department of Medicine, Washington University School of Medicine, St. Louis, MO, United States; 4 Division of Interventional Radiology, Department of Radiology, Washington University School of Medicine, St. Louis, MO, United States

**Keywords:** liver-predominant metastatic uveal melanoma, liver-directed therapy, immunoembolization, melphalan hepatic delivery system, FOCUS trial, progression-free survival, overall survival, disease control rate

## Abstract

**Introduction:**

Approximately 50% of patients with uveal melanoma develop metastatic disease, most commonly involving the liver, and prognosis remains poor. Immunoembolization (IE) is an established liver-directed therapy for patients with liver-dominant metastatic uveal melanoma (mUM), though comparative data with other regional therapies remain limited. We present a single-center retrospective analysis of IE in hepatic mUM and compare outcomes with those reported in the Phase 3 FOCUS trial evaluating melphalan via percutaneous hepatic perfusion.

**Methods:**

All patients with liver-dominant mUM treated with IE between 2010 and 2023 at our institution were included. Clinical records were reviewed for demographics, tumor characteristics, treatment details, and outcomes. The primary endpoint was disease control rate (DCR), defined as complete response (CR), partial response (PR), or stable disease (SD). Secondary endpoints included progression-free survival (PFS) and overall survival (OS), with subgroup analyses based on prior systemic therapy exposure. Descriptive statistics were used for analysis.

**Results:**

Forty-three patients (62.8% female; median age 62 years; all Caucasian) underwent 249 IE procedures (mean 5.8 procedures per patient) over a median treatment duration of 9.1 months. Choroidal primaries accounted for 95.3% of cases, and 90.7% had liver-only metastases. The overall DCR was 27.9% (CR 4.7%, PR 20.9%, SD 2.3%), while 62.8% experienced disease progression. Median PFS was 0.85 months, and median OS was 32.7 months. One- and two-year OS rates were 69.8% and 55.8%, respectively. In subgroup analysis, patients who had received prior systemic therapy (n=12) demonstrated improved outcomes compared with treatment-naïve patients, with longer median PFS (29.9 vs. 7.2 months) and higher 2-year OS (60.0% vs. 47.0%). Compared with outcomes reported in the FOCUS trial, IE demonstrated shorter median PFS (0.85 vs. 9.0 months) and lower DCR (27.9% vs. 73.6%), but longer median OS (32.7 vs. 20.5 months).

**Discussion:**

IE produced overall survival outcomes comparable to hepatic melphalan delivery despite lower radiographic response rates. Notably, patients previously treated with systemic therapy experienced improved outcomes, suggesting a potential sequencing effect. These findings support IE as a viable liver-directed therapy for liver-dominant mUM. Prospective, multicenter trials are warranted to clarify optimal sequencing strategies and enhance therapeutic outcomes.

## 1 Introduction

Uveal melanoma (UM), the most common primary intraocular malignancy in adults in the United States, accounting for approximately 3-5% of all melanomas, with an annual incidence of roughly 6 cases per million individuals (1). Despite effective local control of the primary tumor, nearly 50% of patients develop metastatic disease (mUM) within 10 years of diagnosis, with a striking predilection for hepatic involvement, occurring in approximately 90% of cases (2–4). The development of liver metastases confers a poor prognosis, with reported median overall survival (mOS) of approximately 21 months, previously 12–15 months prior to the approval of tebentafusp and melphalan/Hepatic Delivery System (HDS) (5, 6).

Managing mUM remains challenging, as standard therapies—including liver-directed treatments (LDT) such as transarterial chemoembolization (TACE) and immunoembolization (IE), as well as systemic immunotherapies with checkpoint blockade antibodies or tebentafusp (HLA-A*02:01-positive patients only)—have demonstrated limited efficacy without durable responses or significant survival benefits (7). Systemic immune checkpoint inhibitors, which have transformed outcomes in cutaneous melanoma, have shown modest activity in mUM, likely reflecting the tumor’s low mutational burden and immunologically cold hepatic microenvironment. Among LDTs, IE represents a biologically distinct strategy that combines ischemic tumor control with localized immune stimulation most commonly through transarterial delivery of human granulocyte-macrophage colony-stimulating factor (GM-CSF), with or without interleukin-2 (IL-2) followed by embolic agents such as ethiodized oil or gelatin sponge particles, to induce both tumor ischemia and *in situ* immune activation within hepatic metastases (8). Other liver-directed approaches—including radioembolization with yttrium-90, percutaneous hepatic perfusion with melphalan using the HDS, and isolated hepatic perfusion—have been explored with variable response rates, though durable survival benefits remain inconsistent.

However, Delcath’s recent FOCUS Phase 3 trial demonstrated promising results for the melphalan/HDS in unresectable mUM, reporting an objective response rate (ORR) of 36.3% with a hepatic ORR of 41.8%, DCR of 73.6%, one-year OS of 80%, mOS of 20.5 months, and median progression-free survival (PFS) of 9 months (7). Unlike IE, which relies on immune-mediated mechanisms and is often applied in patients with lower hepatic tumor burden, melphalan/HDS delivers high-dose regional chemotherapy under conditions of temporary hepatic vascular isolation and extracorporeal blood infiltration, enabling greater cytoreduction at the expense of increased procedural complexity and hematologic toxicity. This trial represents a pivotal advancement in the mUM treatment landscape and has renewed interest in liver-confined regional therapies capable of achieving higher intrahepatic drug concentrations while minimizing systemic toxicity. Concurrently, novel investigational strategies—including adoptive T-cell therapies, bispecific T-cell engagers, combination LDT-immunotherapy regimens, and molecularly targeted approaches based on GNAQ/GNA11 and downstream MAPK pathway alterations—are actively under investigation and may further expand therapeutic options in the near future.

Despite these advances, literature on mUM remains scarce due to the disease’s rarity and clinical complexity, underscoring the need for further research. There is no consensus on the optimal treatment approach, particularly regarding the role of LDTs such as IE. The current literature reports inconsistencies regarding treatment modality and outcomes with limited head-head comparisons between immune-based embolization strategies and cytotoxic regional therapies such as melphalan/HDS, thereby constraining definitive conclusions about relative efficacy and patient selection. As a result, treatment selection is frequently individualized and institution-dependent, highlighting the need for comparative effectiveness data to guide evidence-based decision-making.

The present study provides a large, single-center, retrospective analysis of liver-predominant mUM patients treated with IE. By contributing novel insights from a tertiary academic institution, our findings enhance the limited literature on mUM epidemiology, diagnosis, and management. Furthermore, we contextualize out results through direct comparison with outcomes reported in the Delcath FOCUS trial, acknowledging key methodological and patient selection differences, to better delineate the relative roles of immune-based versus cytotoxic liver-directed strategies in contemporary mUM care.

## 2 Methods and materials

### 2.1 Study design

A single-center retrospective review of patients with hepatic involvement from mUM treated at our tertiary academic institution between January 2010 and December 2023 was conducted. This study period was chosen to optimize data capture, given the rarity of mUM. The initial dataset was created using Epic’s SlicerDicer tool, followed by manual chart review to extract detailed demographic, clinical, pathological, molecular, and management/outcome data.

This retrospective study involving human participants was reviewed and approved by the Institutional Review Board of Washington University in St. Louis (IRB #202312065). The requirement for informed consent was waived in accordance with national legislation and institutional requirements because the study involved analysis of existing data with minimal risk to participants. All procedures performed were conducted in accordance with the ethical standards of the institutional and national research committees and with the Declaration of Helsinki.

The IE procedure utilized a total of 1,500 mcg of GM-CSF is mixed with ethiodized oil to form a slurry. Half of the slurry is administered initially, followed by an intraprocedural dose of 2 million units of IL-2, after which the remaining GM-CSF–ethiodized oil mixture is infused. Treatment is delivered in a lobar fashion via the right or left hepatic artery. Imaging assessment was performed after completion of both right and left lobar treatments, which are usually administered at 1-month intervals. Thus, for patients with bilobar diffuse disease, one hepatic lobe was treated first, followed by a 4-week interval before treatment of the contralateral lobe. Imaging, either with CT or MRI abdomen, was obtained after an additional 4-week interval, resulting in radiographic evaluation approximately 12 weeks after the initial treatment.

### 2.2 Study population

Our cohort included male and female mUM patients aged ≥18 with hepatic-predominant mUM who underwent IE during the study period. Patients were selected based on receiving IE, regardless of treatment status (treatment-naïve or prior treatment with immune checkpoint inhibition, chemotherapy, targeted therapy and/or trans arterial chemoembolization). Exclusion criteria included age under 18, absence of standard research consent at our institution (WashU), and those who did not receive IE. In line with the study’s exploratory aim and the intent to compare outcomes with Delcath’s FOCUS trial, no control group was utilized.

### 2.3 Statistics

Statistical analyses were conducted using Microsoft Excel (Microsoft Corp.), employing built-in formula functions supplemented by manual calculations. Measures of central tendency were summarized, and descriptive characteristics were reported as sample sizes, percentages, and proportions. Continuous variables are reported as mean, standard deviation, median, and range (minimum to maximum), while categorical variables are presented as frequencies and percentages. Time-to-event endpoints for OS and PFS were calculated from the date of first IE therapy to death or last follow-up, and date of disease progression, respectively. Disease progression was defined as radiologic progression confirmed on two consecutive CT or MRI imaging and calculated using the first radiologic date of progression. The primary outcome was DCR. Secondary outcomes included OS and PFS at 12 and 24 months. Subgroup analysis assessed differences between patients who were IE-naïve and those pre-treated with prior systemic therapies. DCR, OS, and PFS results were compared to those reported in the Delcath FOCUS trial, with consideration of methodological differences. Descriptive and exploratory statistics were reported.

## 3 Results

### 3.1 Demographics

A total of 43 patients (median age 62 years; range 18-86) were included in the analysis. The majority were female (n=27, 62.8%) and Caucasian, Non-Hispanic (n=43, 100%). Total follow-up (from time of metastatic diagnosis) comprised a median of 54.1 months. Demographic and follow-up data are reported in **Supplementary Table S1** (see supplemental material).

Treatment was well tolerated, with 13 patients in the cohort experiencing adverse effects. These included nausea and vomiting (n=9), mild abdominal pain (n=4), intermittent fatigue (n=3) and access-site hematoma (n=3). All side effects were managed medically and conservatively. There were no treatment-reported deaths.

### 3.2 Clinical characteristics

Most cases were choroidal in origin (n=41, 95.3%) and had hepatic-only metastases (n=39, 90.7%). At the time of first IE, baseline lactate dehydrogenase (LDH)—used as a surrogate for hepatic tumor burden per FOCUS trial conventions—was within the institutional upper limit of normal (≤ULN) in 58.1% of patients (n=25), mildly elevated (>ULN to ≤2×ULN) in 32.6% (n=14), and markedly elevated (>2×ULN) in 9.3% (n=4). The majority of patients were treatment-naïve prior to IE (n=31, 72.1%), while the remainder (n=12, 27.9%) had received prior systemic therapy, including immunotherapy or targeted therapy. No patients received TACE prior to IE, and no patients received melphalan/HDS before or after IE. Following immunoembolization, five patients underwent TACE with either doxorubicin or carmustine (three received carmustine, one received doxorubicin, and one received both agents). Three patients received radioembolization, two received combined chemoembolization and radioembolization, and one patient underwent microwave ablation. SF3B1 mutations were identified in 9.3% of the cohort (n=4).

In total, 249 IE procedures were performed, averaging 5.8 per patient over a mean treatment duration of 9.1 months. Mean LDH at day 1 of initial IE was 308.3 U/L (interquartile range [IQR], 207.5–324.0), compared with 373.6 U/L (IQR, 183.0–341.5) following IE, representing a mean per-patient increase of 29.7%. The resulting DCR after completion of IE was 27.9% (n=12; 4.7% CR, 20.9% PR, and 2.3% SD). Patients with unknown response status were included for baseline and demographic analyses only and were excluded from statistical analyses of DCR, progression-free survival (PFS), and overall survival (OS).

**Figure 1:**
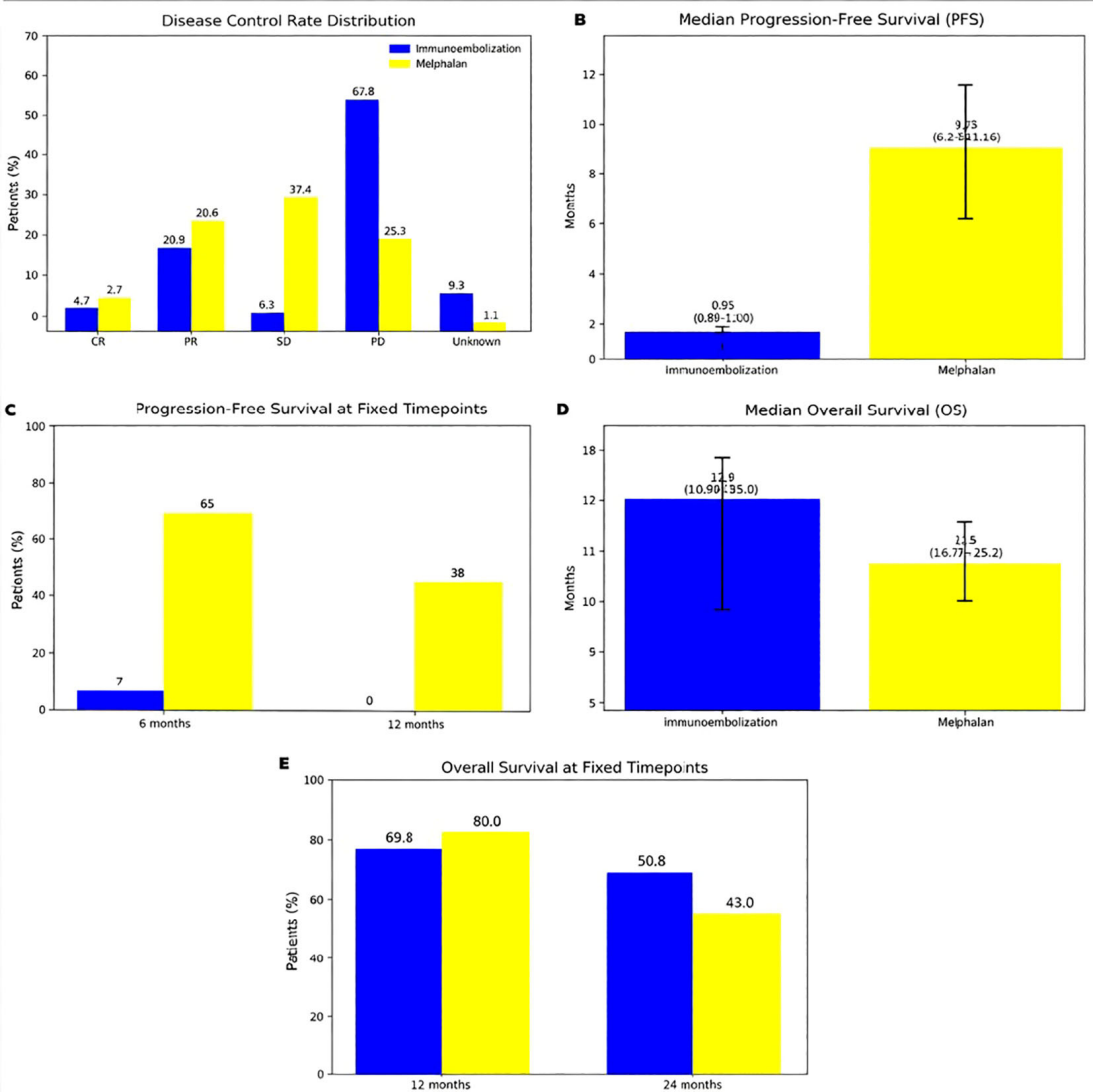
Comparative survival and disease control outcomes between hepatic immunoembolization and melphalan via percutaneous hepatic perfusion. (see **Supplementary Table S1** of the supplemental material). **(A)** Disease control rate (DCR) distribution including complete response (CR), partial response (PR), stable disease (SD), progressive disease (PD), and unknown; **(B)** Median progression-free survival (PFS) with 95% confidence intervals; **(C)** PFS at 6 and 12 months; **(D)** Median overall survival (OS) with 95% confidence intervals; **(E)** OS at 12 and 24 months.

Median PFS for the cohort was 0.85 months, and median OS was 32.7 months. PD occurred in 62.8% of patients (n=27). The estimated 1-year OS was 69.8%, and the 2-year OS was 55.8%. These results are summarized in **Figure 1**. No clinically significant toxicities were attributable to IE.

### 3.3 Subgroup analysis

**Figure 2:**
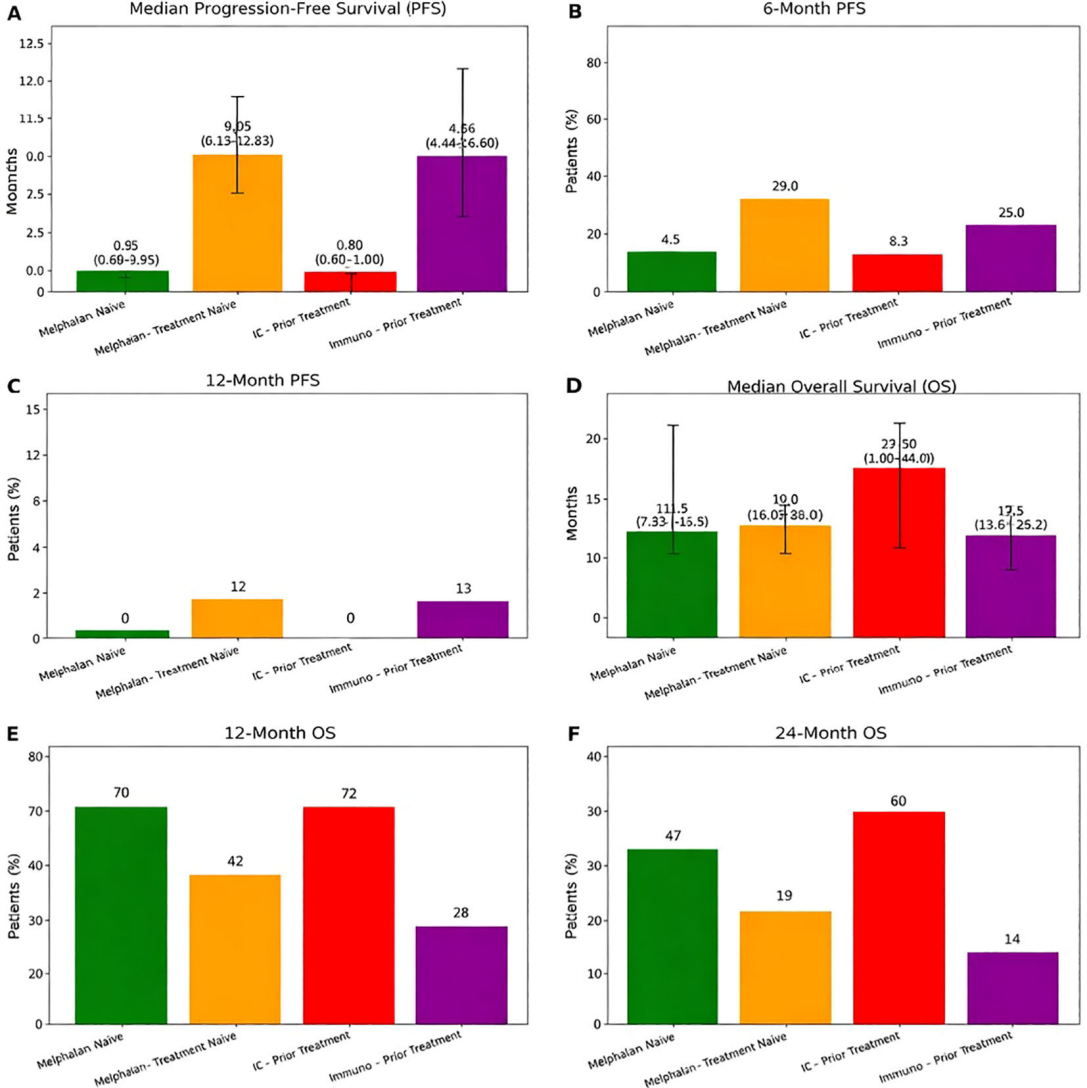
Subgroup survival outcomes stratified by prior treatment status for hepatic immunoembolization and melphalan via percutaneous hepatic perfusion. (see **Supplementary Table S2** of the supplemental material). **(A)** Median progression-free survival (PFS) with 95% confidence intervals; **(B, C)** PFS at 6 and 12 months; **(D)** Median overall survival (OS) with 95% confidence intervals; **(E, F)** OS at 12 and 24 months.

Subgroup analysis compared treatment-naïve patients (n = 31) with those who received prior therapy (n = 12) (**Figure 2**). Baseline LDH at the time of first IE did not differ significantly between groups (median 240 U/L vs. 301 U/L, p = 0.616). Following IE, LDH values remained statistically comparable (median 238 U/L vs. 207 U/L, p = 0.273), although treatment-naïve patients demonstrated a greater mean per-patient increase in LDH compared with previously treated patients (+44.0% vs. −7.3%); this difference did not reach statistical significance (p = 0.218). SF3B1 mutation frequency was similar between groups (6.5% vs. 16.7%, p = 0.308).

Interestingly, the previously treated group demonstrated a longer median PFS (29.9 months vs. 7.2 months) and a higher complete response rate (8.3% vs. 3.2%). Overall, DCR was comparable between groups: 29% in the treatment-naïve patients (3.2% CR, 22.6% PR, 3.2% SD) versus 25% in the previously treated patients (8.3% CR, 16.7% PR). While objective response rate (ORR) [CR+PR] appeared higher in previously treated patients (66.7% vs. 32.3%), this difference did not meet statistical significance (p=0.082). Survival outcomes favored the previously treated cohort, with higher estimated 1-year OS (72.0% versus 70.0%) and 2-year OS (60.0% versus 47.0%).

Subgroup outcomes were further compared with those reported in the FOCUS trial (**Figure 2**). Treatment-naïve IE patients demonstrated a lower DCR (29%) compared with treatment-naïve patients in the FOCUS trial (80.4%; 5.9% CR, 29.4% PR, 45.1% SD) (7). A similar pattern was also noticed when comparing the DCR in the previously treated IE group (25% DCR; 8.3% CR, 16.7% PR) and the previously treated FOCUS trial’s group (65% DCR; 10% CR, 27.5% PR, 27.5% SD) (7). Despite lower DCRs in both IE groups, median PFS was similar between the treatment-naïve groups (7.2 months for IE vs. 9.0 months in the FOCUS trial) but was notably longer in the pretreated IE group (29.9 months) compared to the pretreated FOCUS group (9.18 months) (7). Median OS was also comparable in the treatment naïve groups (21.6 months for IE vs 20.5 months in the FOCUS trial) (7). However, the median OS was higher in the pretreated IE group (35.5 months) compared to the pretreated FOCUS trial group (20.8 months) (7).

When stratified by response status, responders (n=2) demonstrated no improvement in median PFS compared with non-responders (0 vs 0.89 months), yet experienced a longer median OS from first IE (34.9 vs 32.7 months), highlighting a discordance between radiographic response and survival outcomes.

## 4 Discussion

Nearly 50% of individuals with UM eventually develop metastatic disease, with liver involvement in approximately 90% of cases (2–4). Hepatic metastasis is associated with poor prognosis, highlighting a critical clinical and management gap for this vulnerable population (5). Despite advances, therapeutic options for mUM remain limited and often have minimal impact on prognosis. Tebentafusp has shown a meaningful 1-year OS rate of 73% and a 3-year OS of 27% in HLA–A*02:01 positive patients, despite a low ORR (9, 10). However, its utility is notably restricted to HLA–A*02:01 positive individuals. Immune checkpoint inhibitors, whether as monotherapy or in combination regimens, have demonstrated modest activity, with 1-year OS rates of 52-56% and low ORRs (9, 11). Liver-directed therapies such as TACE, IE, and radioembolization remain essential for managing liver-dominant disease, yet definitive evidence for durable survival benefit is lacking (12–14).

**Figure 3:**
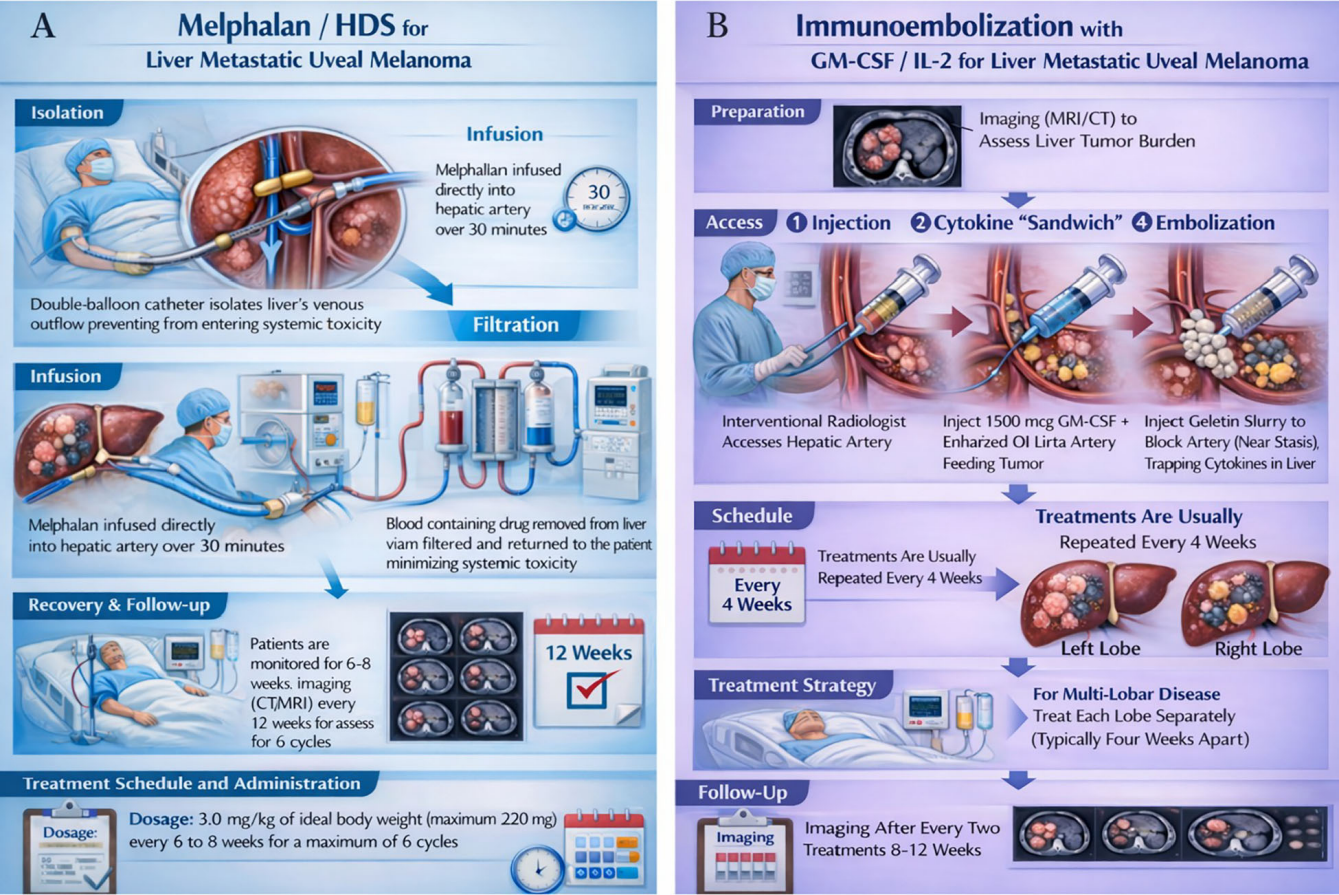
**(A)** Melphalan/HDS therapy for liver metastatic uveal melanoma and **(B)** Immunoembolization with GM-CSF/IL-2 therapy.

Melphalan/HDS and IE with GM-CSF ± IL-2 and Gelfoam represent two fundamentally different liver-directed strategies, with distinct procedural complexity and toxicity profiles. Melphalan/HDS is a technically complex, whole-liver treatment in which hepatic venous outflow is isolated using a double-balloon catheter in the inferior vena cava, melphalan is infused via the hepatic artery, and hepatic venous blood is diverted through an extracorporeal filtration circuit before systemic return; treatments are typically administered every 6–8 weeks for a limited number of cycles and require intensive peri-procedural monitoring (**Figure 3A**). In the FOCUS trial, most common adverse events were thrombocytopenia (15.8%) and neutropenia (10.5%) (7). No treatment-related deaths were observed (7). In contrast, IE with GM-CSF ± IL-2 is a selective arterial embolization-based approach, typically performed on a lobar basis, in which immune-stimulating cytokines (often delivered in ethiodized oil) are infused into tumor-feeding hepatic arterial branches followed by gelatin sponge embolization to induce ischemia and local immune activation; treatments are commonly repeated every 4 weeks with alternating lobes for bi-lobar disease (**Figure 3B**). Accordingly, the toxicity profile of IE is driven primarily by post-embolization syndrome, including fever, right upper quadrant abdominal pain, nausea, fatigue, and transient transaminase elevations, with severe or prolonged abdominal pain and higher-grade hepatic toxicity occurring less frequently. In this single-institution study, 13 patients experienced adverse effects, including nausea and vomiting (20.9%), mild abdominal pain (9.3%), intermittent fatigue (6.98%), and access-site hematoma (6.98%). Similar to the FOCUS trial, side effects were managed medically and conservatively with no treatment-reported deaths.

Delcath’s recent multicenter, open-label, single-arm phase 3 FOCUS trial offered promising results for the melphalan/Hepatic Delivery System in patients with unresectable mUM. Eligible patients (N = 91) received melphalan at a dose of 3.0 mg/kg (ideal body weight) every 6–8 weeks, for up to six cycles (7). Like our design, the primary endpoint was ORR, with secondary endpoints including duration of response, OS, and PFS (7). The study demonstrated an ORR of 36.3%, 1-year OS of 80.0%, mOS of 20.5 months, and median PFS of 9 months (7).

In contrast, our single-institution retrospective cohort treated with IE demonstrated lower radiographic response rates, with a DCR of 27.9% and an ORR of 25.6% across the entire cohort. Despite this, survival outcomes were broadly comparable to those reported in the FOCUS trial. Median PFS in our cohort was 0.85 months, and median OS was 32.7 months, with 1- and 2-year OS rates of 69.8% and 55.8%, respectively. These findings suggest that although IE is associated with fewer objective radiographic responses than melphalan-based hepatic perfusion, it may nonetheless confer durable disease control and clinically meaningful survival in a real-world setting.

Subgroup analyses further refine these observations. When stratified by prior treatment exposure, treatment-naïve patients receiving IE demonstrated a median PFS of 7.2 months and median OS of 21.6 months—closely mirroring outcomes observed in treatment-naïve patients enrolled in the FOCUS trial (median PFS 9.0 months; median OS 20.5 months) (7). However, response rates in treatment-naïve IE patients were substantially lower than those reported in FOCUS (DCR 29% vs 80.4%), highlighting differences in radiographic response despite similar survival outcomes (7). Notably, exploratory analyses stratified by responder status demonstrated that patients classified as responders experienced longer OS compared with non-responders (median OS 34.9 vs 32.7 months), despite no corresponding prolongation in median PFS (0.0 vs 0.9 months). These findings suggest that radiographic response category may retain prognostic relevance for survival, even as conventional PFS metrics incompletely capture clinical benefit in this heterogeneous cohort.

In the FOCUS trial, baseline LDH was also used as a surrogate for hepatic tumor burden and dichotomized as normal/low versus elevated relative to the ULN. Approximately 60% of treated patients had normal or low LDH at baseline, while ~40% had elevated LDH, with LDH demonstrating strong prognostic significance. Patients with normal/low LDH experienced significantly longer overall survival compared with those with elevated LDH (median OS ~23.5 vs ~15.3 months). In our IE cohort, baseline LDH distribution closely mirrored that reported in FOCUS when assessed at the time of first IE using comparable ULN-based conventions. LDH was within normal limits (≤ULN) in 58.1% of patients, mildly elevated (>ULN to ≤2×ULN) in 32.6%, and markedly elevated (>2×ULN) in 9.3%, resulting in 41.9% of patients classified as having elevated LDH. Mean LDH increased from 308.3 U/L (IQR 207.5–324.0) at day 1 of initial IE to 373.6 U/L (IQR 183.0–341.5) following treatment, representing a mean per-patient increase of 29.7%, consistent with expected post-embolization hepatocellular injury rather than interval disease progression. LDH kinetics in our cohort did not significantly differ between treatment-naïve and pretreated patients, suggesting that disease burden alone does not fully explain observed differences in response.

Interestingly, patients who had received prior systemic or liver-directed therapy before IE demonstrated numerically superior outcomes compared with both treatment-naïve IE patients and the pretreated cohort in the FOCUS trial. In our study, previously treated patients achieved a median PFS of 29.9 months and median OS of 35.5 months, compared with a median PFS of 9.18 months and median OS of 20.8 months in the pretreated FOCUS population (7). Although IE demonstrated substantially lower disease control rates than melphalan/HDS, the observation of a longer overall survival suggests that radiographic response captures benefit in a subset of patients, but median cohort-level response metrics may underestimate the impact of durable responders. Additionally, OS may represent a more appropriate benchmark for comparing liver-directed therapies in metastatic uveal melanoma. Furthermore, while subgroup analyses were underpowered and differences did not reach statistical significance, the findings raise the possibility that prior therapy may favorably modulate tumor biology or the hepatic immune microenvironment, thereby enhancing responsiveness to IE. Alternatively, these observations may reflect selection bias, as patients able to receive sequential therapies may constitute a biologically favorable subset with more indolent disease progression.

The SCANDIUM trial was a phase 3 randomized controlled study that evaluated isolated hepatic perfusion (IHP) with melphalan versus best alternative care (BAC) in patients with previously untreated, isolated UM with liver metastases (15). BAC included chemotherapy, immune checkpoint inhibitors, and other locoregional therapies (15). In this study, IHP significantly improved PFS (median 7.4 versus 3.3 months; hazard ratio 0.21) and ORR (40% versus 45%) compared to BAC (15). However, the primary end point of OS at 24 months was not met. 24-month OS was 46.5% for IHP versus 29.5% for BAC (P = 0.12), and median OS was 21.7 vs 17.6 months (HR 0.64, 95% CI 0.37–1.10) (15). Collectively, both the SCANDIUM and FOCUS trials demonstrated that regional melphalan-based liver-directed therapy yields high response rates and median OS of approximately 20–22 months, with manageable toxicity profiles (7, 15). Both trials showed manageable toxicity profiles. Nevertheless, no standardized treatment algorithm for mUM currently exists, and real-world comparative data across liver-directed modalities remain limited.

We present a single-institution retrospective analysis of liver-predominant mUM treated with IE, providing critically relevant real-world outcome data enabling comparison with Delcath’s FOCUS trial (**Figure 1** and **2**). While response rates in our cohort were lower than those in the FOCUS and SCANDIUM trials, median PFS and OS were comparable to FOCUS and exceeded those reported in SCANDIUM. Notably, previously treated patients receiving IE demonstrated longer median PFS and OS than pretreated patients in the FOCUS trial, suggesting potential benefit in selected populations.

Prior studies further support the durability of IE. A randomized phase 2 trial demonstrated delayed (N = 53) systemic progression in liver-dominant mUM and a median OS 21.5 months with IE, consistent with outcomes observed in our cohort (16). Our study data demonstrated a similar PR of 20.9% but unlike Valsechhi et al., our data demonstrated CR (4.7%). A large real-world analysis from Thomas Jefferson University reported a median OS of 20 months from IE initiation, with 1-, 2-, 3-, and 5-year OS of 73.2%, 41.8%, 25.3%, and 11.2%, respectively (17). Although our 1-year OS rate was slightly lower (69.8%), the median OS was higher (32.7months) (17). Unlike some prior series, our cohort also demonstrated complete responses following IE, albeit infrequently.

Taken together, these findings suggest that while melphalan-based hepatic perfusion, as evaluated in the FOCUS and SCANDIUM trials, achieves higher radiographic response rates, IE may provide comparable survival outcomes with potentially greater accessibility and lower procedural complexity. DCRs reported for IE have varied across published studies, with some series demonstrating higher DCRs comparable to those observed with melphalan/HDS in the FOCUS trial, while achieving overall survival outcomes similar to those reported in our cohort. The discordance between response rates and survival observed across studies reinforces the notion that radiographic response may not fully capture the clinical benefit of liver-directed therapies in mUM.

Several limitations warrant consideration. As a single-center retrospective study, our findings may not be fully generalizable and are subject to selection bias and unmeasured confounding. Cross-trial comparisons, including those with the FOCUS trial, are inherently limited by differences in study design, patient selection, disease burden, prior treatment exposure, and follow-up. Additionally, prognostically relevant variables such as baseline LDH levels, M1 substage, and extent of hepatic tumor involvement were not uniformly available across studies and could not be fully harmonized. Accordingly, these findings should be interpreted as hypothesis-generating rather than definitive.

## 5 Conclusion

We present our tertiary center’s unique experience via one of the largest retrospective analyses to date, evaluating IE in liver-predominant mUM. By directly comparing our outcomes to those of the Delcath FOCUS trial, we offer a novel perspective on the real-world efficacy of IE, particularly in a cohort that includes both treatment-naïve and previously treated patients. While no standardized treatment approach exists for mUM, our findings suggest that IE offers PFS and OS outcomes comparable to those achieved with the melphalan/HDS, with a potential survival advantage in patients with prior systemic therapy. These results contribute important insights into the evolving landscape of liver-directed therapies and underscore the value of individualized, multidisciplinary treatment approaches. While we offer unique insights and advance the understanding of mUM diagnosis/management, the literature remains limited—larger, prospective studies are needed to refine patient risk-stratification and for future therapeutic strategies to optimize patient care.

## Data Availability

The original contributions presented in the study are included in the article/supplementary material. Further inquiries can be directed to the corresponding author.
