# Supplementary material for: Immunoembolization in liver-predominant metastatic uveal melanoma: a single-center retrospective analysis
Source: Front Oncol. 2026 Apr 23;16:1752725. doi: 10.3389/fonc.2026.1752725 (PMC13149174; doi:10.3389/fonc.2026.1752725)
Supplement: Supplementary file 1 [file Table1.docx]

Immunoembolization in Liver-Predominant Metastatic Uveal Melanoma: A Single-Center Retrospective Analysis

**Renee Morecroft MD^1^, Jordan Phillipps MD^4^, Amrat Kumar MD^2^, Jacob Strelnikov MD^2^, George Nassief BA^2^, Naganathan Mani MD^3^, Jennifer Gould MD^3^, Tanner Johanns MD, PhD^2^, George Ansstas MD^2*^**

^1^Department of Internal Medicine, HCA Florida Orange Park Hospital, Orange Park, FL, USA

^2^Division of Medical Oncology, Department of Medicine, Washington University School of Medicine, St. Louis, MO, USA

^3^Division of Interventional Radiology, Department of Radiology, Washington University School of Medicine, St. Louis, MO, USA

^4^Department of Dermatology, Mayo Clinic Florida, Jacksonville, FL, USA

Supplementary Material

Supplementary Tables

**Table 1:** Comparing overall data for hepatic immunoembolization and melphalan via percutaneous hepatic perfusion

|  | | **Hepatic Immunoembolization** | **Melphalan via Percutaneous Hepatic Perfusion** |
| --- | --- | --- | --- |
| **Total # of Participants** | | 43 | 91 |
| **Age** | | | |
| Median, years (range) | | 62.0 (18-86) | 61 (20-78) |
| <65, n (%) | | 23 (53.5) | 61 (67.0) |
| >65, n (%) | | 20 (46.5) | 30 (33.0) |
| **Sex** | | | |
| Female, n (%) | | 27 (62.8) | 47 (51.6) |
| Male, n (%) | | 16 (37.2) | 44 (48.4) |
| **Ethnicity** | | | |
| Hispanic, n (%) | | 0 | 2 (2.2) |
| Non-Hispanic, n (%) | | 43 (100) | 86 (94.5) |
| Unknown, n (%) | | 0 | 3 (3.3) |
| **Race** | | | |
| White, n (%) | | 43 (100) | 86 (94.5) |
| Other, n (%) | | 0 | 2 (2.2) |
| Unknown, n (%) | | 0 | 3 (3.3) |
| **Presence of extrahepatic lesions** | | | |
| Hepatic only, n (%) | | 39 (90.7) | 64 (70.3) |
| Extrahepatic, n (%) | | 4 (9.3) | 27 (29.7) |
| **Lactose Dehydrogenase** | | | |
| Normal /Low (≤ ULN) | | 25 (58.1) | 55 (~60%) |
| Elevated: >ULN to ≤ 2× ULN | | 14 (32.6) | 35 (~40%) |
| Elevated: >2× ULN | | 4 (9.3%) |  |
| **Prior Therapy** | | | |
| Naive, n (%) | | 31 (72.1) | 51 (56.0) |
| Previously Treated, n (%) | Overall | 12 (27.9) | 40 (44.0) |
|  | Radiation | 0 | 10 (11.0) |
|  | Surgery | 0 | 13 (14.3) |
|  | Immunotherapy and targeted therapy | 12 (27.9) | 23 (25.3) |
| **Disease Control Rate, n (%)** | | | |
| Complete Response | | 2 (4.7) | 7 (7.7) |
| Partial Response | | 9 (20.9) | 26 (28.6) |
| Stable Disease | | 1 (2.3) | 34 (37.4) |
| Progressive Disease | | 27 (62.8) | 23 (25.3) |
| Unknown | | 4 (9.3) | 1 (1.1) |
| **Progress Free Survival** | | | |
| median (95% CI) | | 0.85 (0.69-0.95) | 9.0 (6.34-11.56) |
| 6 months, % | | 7 | 65 |
| 12 months % | | 0 | 38 |
| **Overall Survival** | | | |
| median (95% CI) | | 32.7 (15.9-38.5) | 20.5 (16.79-25.26) |
| 12 months, % | | 69.8 | 80 |
| 24 months, % | | 55.8 | 43 |
