# Supplementary material for: Immunoembolization in liver-predominant metastatic uveal melanoma: a single-center retrospective analysis
Source: Front Oncol. 2026 Apr 23;16:1752725. doi: 10.3389/fonc.2026.1752725 (PMC13149174; doi:10.3389/fonc.2026.1752725)
Supplement: Supplementary file 2 [file Table2.docx]

Immunoembolization in Liver-Predominant Metastatic Uveal Melanoma: A Single-Center Retrospective Analysis

**Renee Morecroft MD^1^, Jordan Phillipps MD^4^, Amrat Kumar MD^2^, Jacob Strelnikov MD^2^, George Nassief BA^2^, Naganathan Mani MD^3^, Jennifer Gould MD^3^, Tanner Johanns MD, PhD^2^, George Ansstas MD^2*^**

^1^Department of Internal Medicine, HCA Florida Orange Park Hospital, Orange Park, FL, USA

^2^Division of Medical Oncology, Department of Medicine, Washington University School of Medicine, St. Louis, MO, USA

^3^Division of Interventional Radiology, Department of Radiology, Washington University School of Medicine, St. Louis, MO, USA

^4^Department of Dermatology, Mayo Clinic Florida, Jacksonville, FL, USA

Supplementary Material

Supplementary Tables

**Table 2:** Comparing subgroup analysis for hepatic immunoembolization and melphalan via percutaneous hepatic perfusion.

| **Subgroups** | **Treatment Naïve Hepatic Immunoembolization** | **Treatment Naïve Melphalan via Percutaneous Hepatic Perfusion** | **Hepatic Immunoembolization with Prior Treatment** | **Melphalan via Percutaneous Hepatic Perfusion with Prior Treatment** |
| --- | --- | --- | --- | --- |
| **Total Number of Participants** | 31 (72.1) | 51 (56) | 12 (27.9) | 40 (44) |
| **Age** | | |  |  |
| Baseline (mean, years) | 62.9 | 58.1 | 54.9 | 61.7 |
| **Sex** | | |  |  |
| Female, n (%) | 65% | 49% | 58% | 60% |
| Male, n (%) | 35% | 51% | 42% | 40% |
| **Time since liver metastatic diagnosis** | | |  |  |
| Months, median (range) | 54.1 (45.8-62.5) | 5.7 (0.2-109.3) | 50.4 (40.3-60.3) | 2.5 (0.4-26.0) |
| **Disease Control Rate, n (%)** | | |  |  |
| Complete Response | 1 (3.2) | 3 (5.9) | 1 (8.3) | 4 (10.0) |
| Partial Response | 7 (22.6) | 15 (29.4) | 2 (16.7) | 11 (27.5) |
| Stable Disease | 1 (3.2) | 23 (45.1) | 0 | 11 (27.5) |
| Progressive Disease | 20 (64.5) | 10 (19.6) | 7 (58.3) | 13 (32.5) |
| Unknown | 2 (6.5) | 0 | 2 (16.7) | 1 (2.5) |
| **Progress Free Survival** | | |  |  |
| Median (95% CI) | 0.85 (0.66-0.95) | 9.0 (6.11-12.81) | 0.80 (0.00-1.08) | 9.18 (4.44-14.06) |
| 6 months, % | 6.5 | 29 | 8.3 | 25 |
| 12 months % | 0 | 12 | 0 | 13 |
| **Overall Survival** | | |  |  |
| Median (95% CI) | 21.6 (18.7-43.2) | 20.5 (16.7-28.2) | 35.5 (7.8-44.4) | 20.8 (14.0-26.7) |
| 12 months, % | 70 | 42 | 72 | 28 |
| 24 months, % | 47 | 19 | 60 | 14 |
